# Octanoic Acid‐Rich Enteral Nutrition Alleviates Sepsis‐Associated Metabolic Disorders by Suppressing White Adipose Tissue Browning in Mice

**DOI:** 10.1002/fsn3.72020

**Published:** 2026-06-09

**Authors:** Chunliang Zheng, Xiaohua Li, Chungen Xing, Chun Cao

**Affiliations:** ^1^ Department of General Surgery The Second Affiliated Hospital of Soochow University Suzhou China; ^2^ Department of Thyroid and Breast Surgery Suzhou Wuzhong People's Hospital Suzhou China

**Keywords:** enteral nutrition, lactate, octanoic acid, sepsis, white adipose tissue browning

## Abstract

Sepsis is a life‐threatening clinical syndrome accompanied by severe systemic metabolic disorders, and pathological white adipose tissue (WAT) browning is a core driver that exacerbates negative energy balance, skeletal muscle dysfunction and insulin resistance. Octanoic acid (OA), a C8 medium‐chain fatty acid with superior ketogenic capacity, is a promising functional nutrient for sepsis intervention. This study aimed to investigate the protective effects and underlying mechanism of OA‐rich enteral nutrition (EN) on sepsis‐associated metabolic disorders. C57BL/6 mice were subjected to lipopolysaccharide (LPS) to establish sepsis models and received OA‐rich EN with different concentrations; pharmacological activation of GPR81 and genetic activation of CTRP6 and p38 were used to verify the GPR81‐CTRP6‐p38 pathway. Results revealed that OA‐rich EN dose‐dependently increased serum β‐hydroxybutyrate levels and decreased lactate accumulation in serum and WAT, with markedly better efficacy than conventional EN. OA‐rich EN significantly inhibited LPS‐induced upregulation of GPR81, CTRP6, phosphorylated p38 and UCP1 in WAT, mitigated WAT atrophy and abnormal browning, thereby alleviating excessive energy expenditure, body weight loss, skeletal muscle dysfunction and insulin resistance. Mechanistically, activation of GPR81, CTRP6 or p38 to promote WAT browning reversed the protective effects of OA‐rich EN, confirming that OA‐rich EN alleviated metabolic disorders by suppressing WAT browning via blocking the lactate‐GPR81‐CTRP6‐p38 pathway. In conclusion, OA‐rich EN enhanced hepatic ketogenesis to reduce lactate accumulation, thereby inhibiting the GPR81‐CTRP6‐p38 pathway and suppressing pathological WAT browning, ultimately alleviating sepsis‐associated metabolic disorders. These findings provide a novel targeted nutritional strategy and theoretical basis for optimizing clinical nutritional support in septic patients.

## Graphical abstract

Octanoic acid‐rich enteral nutrition enhances hepatic ketogenesis to decrease lactate accumulation in septic mice. This further blocks the lactate‐GPR81‐CTRP6‐p38 pathway, thereby suppressing pathological white adipose tissue browning and its subsequent hypermetabolic effects. Consequently, octanoic acid‐rich enteral nutrition alleviates energy imbalance, skeletal muscle dysfunction, and insulin resistance, providing a novel targeted nutritional intervention strategy for the clinical management of sepsis‐associated systemic metabolic disorders.

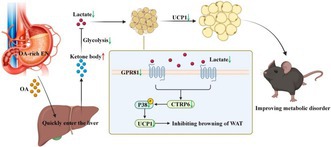

## 1 Introduction

Sepsis is a life‐threatening clinical syndrome caused by a dysregulated host immune response to infection, which is accompanied by severe systemic metabolic disorders that significantly worsen patient prognosis and elevate mortality (Kox et al. 2026). In the septic stress state, the body undergoes a series of pathological metabolic alterations, including anorexia, excessive glycolysis activation, accelerated skeletal muscle proteolysis, and insulin resistance; among these, insulin resistance impairs glucose utilization efficiency, thereby forming a vicious cycle of metabolic imbalance (Isidor et al. 2022; Tang et al. 2025). Additionally, sepsis‐induced dyslipidemia and persistent systemic inflammation further exacerbate metabolic disorders, impairing multiple organ functions and hindering the recovery of critically ill patients (Grondman et al. 2020; Hofmaenner et al. 2022). These intractable metabolic disorders have become a major bottleneck in the clinical treatment of sepsis, making the exploration of targeted regulatory mechanisms and effective intervention strategies an urgent clinical requirement.

Nutritional support is an indispensable cornerstone of sepsis therapy, and enteral nutrition (EN) is recognized as the preferred nutritional intervention route for septic patients due to its advantages in preserving intestinal mechanical, immune and biological barrier functions, regulating immune responses, and reducing infectious complications (Wischmeyer et al. 2023). However, landmark clinical studies including EPaNIC and EDEN have demonstrated that traditional full‐dose EN or early parenteral nutrition fails to effectively alleviate the metabolic disorders in septic patients, and excessive nutritional supplementation may even increase the burden on hepatic and renal functions and elevate the risk of complications and mortality (Casaer et al. 2011; Hermans et al. 2013). This is mainly attributed to the blunted responsiveness to exogenous nutrition caused by severe metabolic disorders during the septic stress period, which indicates that optimizing the EN formula and exploring targeted nutritional intervention strategies based on the underlying mechanisms of sepsis‐associated metabolic disorders is the key to improving the efficacy of clinical nutritional support.

Octanoic acid (OA), a C8 medium‐chain fatty acid (MCFA), is naturally present in coconut oil and dairy products (Lemarié et al. 2018). OA has emerged as a promising nutritional supplement for sepsis treatment due to its unique metabolic characteristics and biological activities. Unlike long‐chain fatty acids, which rely on the lymphatic system for absorption and carnitine for transport during metabolism, OA is rapidly absorbed via the portal vein and directly metabolized in the liver, and it exhibits the highest ketogenic efficiency among all MCFAs (Harvey et al. 2018; Vandenberghe et al. 2017). OA acts as a direct and efficient substrate for ketone bodies synthesis via the hepatic mitochondrial ketogenic pathway (Lemarié et al. 2016). Ketone bodies can serve as an alternative energy supply for vital organs including the heart and lungs, attenuate systemic inflammation and protect against sepsis‐induced multiple organ dysfunction, which is significant for septic patients with impaired glucose metabolism and insufficient energy supply (Soni et al. 2022). Furthermore, previous studies have demonstrated that OA‐rich EN exerts multiple protective effects in sepsis, including anti‐inflammatory and immunoregulatory effects, and attenuation of acute hepatic and intestinal injury, suggesting that OA‐rich EN is a multifaceted nutritional intervention beyond simple energy supplementation (Tang et al. 2023; Xue and Cao 2025; Zhang et al. 2024; Zhou et al. 2026).

White adipose tissue (WAT) browning is a key pathological event driving metabolic disorders in sepsis (Li et al. 2021). Pathological WAT browning induced by septic stress is defined as the acquisition of brown adipocyte‐like features in WAT, characterized by the formation of smaller multilocular adipocytes, upregulated expression of the thermogenic marker uncoupling protein 1 (UCP1), and increased mitochondrial density and respiratory capacity (Ayalon et al. 2018). Extensive WAT browning dissipates energy as heat, which further exacerbates the hypermetabolic state and negative energy balance in septic patients (Abdullahi and Jeschke 2017). Hyperlactatemia is a well‐recognized predictor of poor prognosis in sepsis (Vincent and Bakker 2021). Excessive lactate accumulation in serum and adipose tissue is caused by sepsis‐induced hyperactivation of glycolysis and glucocorticoid resistance, which impairs hepatic lactate clearance (Vandewalle et al. 2021; Yang et al. 2022). Recent studies have demonstrated that lactate can induce WAT browning and lipolysis, and exacerbate hypermetabolism in various stress states including burns and cachexia (Barayan et al. 2023; Liu et al. 2024). GPR81, a lactate‐specific receptor predominantly expressed in adipose tissue, is the key mediator of lactate‐induced WAT browning (Yao et al. 2020). Lactate binding to GPR81 upregulates the expression of C1q/tumor necrosis factor‐related protein 6 (CTRP6), an adipokine highly homologous to adiponectin, which then rapidly activates p38 phosphorylation and the release of inflammatory factors (Xu et al. 2024). P38 phosphorylation is an essential step for *UCP1* gene transcription, as it promotes the nuclear translocation of ATF2, thereby significantly increasing UCP1 protein expression (Cao et al. 2004).

In this study, we aimed to compare the protective effects of OA‐rich EN with conventional EN against sepsis‐associated metabolic disorders, with a focus on the high ketogenic activity of OA as the core mechanism. We further sought to verify that OA‐mediated ketogenesis was a key mechanism through which OA‐rich EN alleviated sepsis‐associated metabolic disorders through inhibiting lactate accumulation, blocking the GPR81‐CTRP6‐p38 pathway, and ultimately suppressing abnormal WAT browning in septic mice.

## 2 Materials and Method

### 2.1 Chemicals and Reagents

The commercial EN solution (Ensure Nutrison) was obtained from Abbott (USA). OA (CAS number: 124‐07‐2, purity ≥ 99%) was obtained from Aladdin (China). Lipopolysaccharide (LPS, CAS number: 93572–42‐0) was obtained from Sigma‐Aldrich (USA). 3‐chloro‐5‐hydroxybenzoic acid (CHBA, CAS number: 53984‐36‐4) was obtained from MedChemExpress (China). Adeno‐associated virus serotype 9 (AAV9)‐adiponectin‐GFP‐CTRP6 mus and AAV9‐adiponectin‐GFP‐MKK6 mus were obtained from GenePharma (China). The lactate assay kit (Cat number: KTB1100) was obtained from Abbkine (China). The insulin ELISA kit (Cat number: ELM‐Insulin‐1) was obtained from RayBiotech (USA). UCP1 (Cat number: 83870) and β‐tubulin (Cat number: 10094) antibodies were obtained from Proteintech (China). GPR81 (Cat number: NBP1‐51956) antibody was obtained from Novus Biologicals (USA). CTRP6 (Cat number: AB36900) antibody was obtained from Abcam (UK). p38 (Cat number: 8690) and p‐p38 (Cat number: 4511) antibodies were obtained from Cell Signaling Technology (USA).

### 2.2 Animals

Six‐ to eight‐week‐old male C57BL/6 mice (weighing 19.5–26.0 g) were obtained from Soochow University. All mice were housed under specific pathogen‐free conditions with a 12 h light/dark cycle, controlled temperature (22°C ± 2°C), and relative humidity (50% ± 5%). Mice had free access to standard chow and water for a 1‐week acclimatization period prior to the experiment. The experimental protocols were approved by the Institutional Animal Care and Use Committee of Soochow University.

### 2.3 Placement of Enteral Feeding Tubes

Gastric tube placement was performed in all mice as previously described (Cao et al. 2020). Briefly, a medical‐grade silicone rubber tube (Helix Medical, Silastic 60‐011‐03, Germany) was distally inserted into the gastric cavity via a puncture in the gastric corpus using a thick needle and fixed at the gastric corpus with a purse‐string suture. During the 72‐h surgical recovery period, all mice received 0.9% saline via the gastric tube.

### 2.4 Experimental Protocols

**FIGURE 1:**
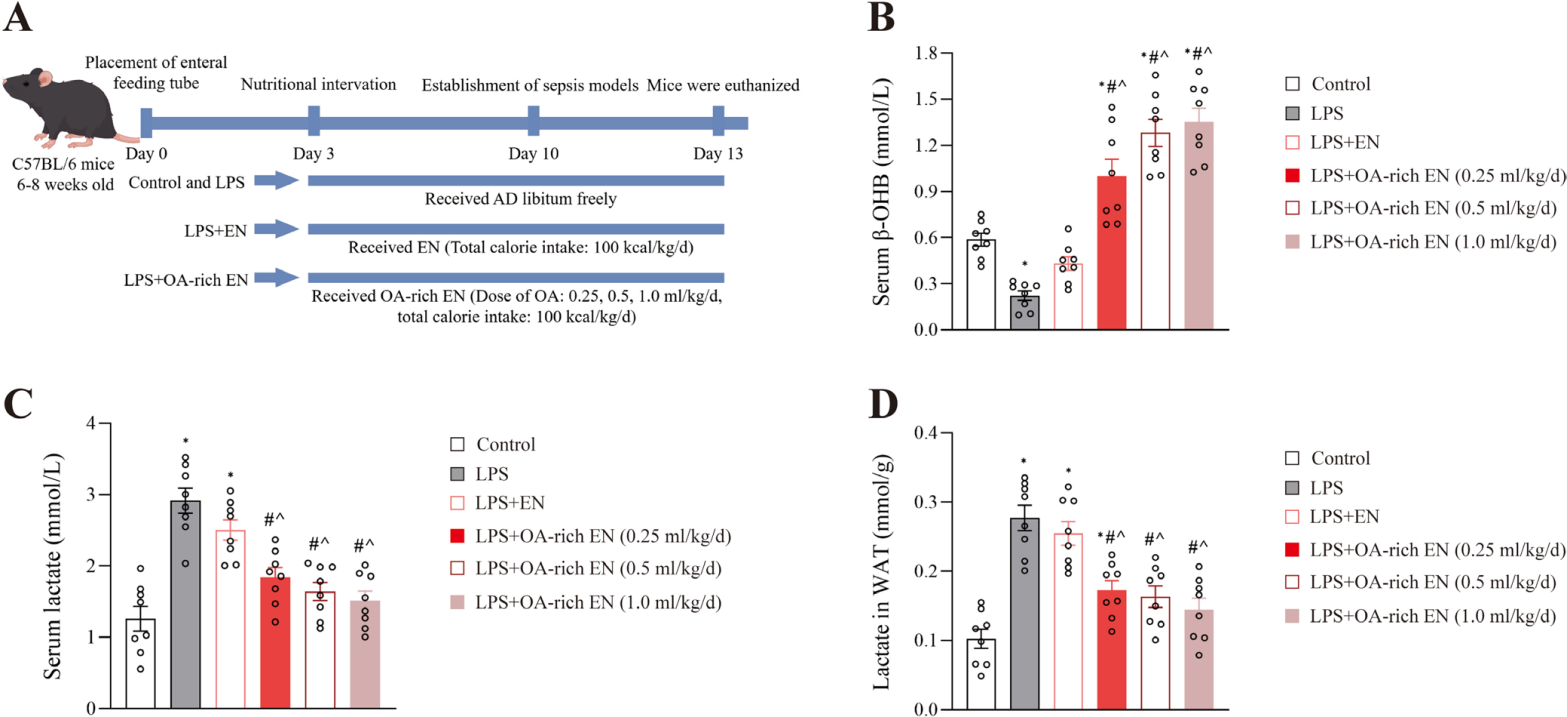
OA‐rich EN enhanced ketogenesis and decreased lactate accumulation in serum and WAT of septic mice. Schematic illustration of the experimental design (A). Serum β‐OHB (B) was measured using a ketone meter. Lactate in serum (C) and WAT (D) were measured using a lactate assay kit. *N* = 8 per group. Data are presented as the mean ± standard error of the mean. *p < 0.05 versus the Control group. #*p* < 0.05 versus the LPS group. ^*p* < 0.05 versus the LPS + EN group. EN, enteral nutrition; LPS, lipopolysaccharide; OA, octanoic acid; WAT, white adipose tissue; β‐OHB, β‐hydroxybutyrate.

First, to investigate the effects of OA‐rich EN at different concentrations on metabolic disorders in septic mice, mice were randomly divided into six groups (*n* = 8 per group): Control, LPS, LPS + EN, LPS + OA‐rich EN (0.25 mL/kg/day), LPS + OA‐rich EN (0.5 mL/kg/day), and LPS + OA‐rich EN (1.0 mL/kg/day). Mice in Control and LPS groups received standard food and water, whereas those in the LPS + EN group received a commercial EN solution via the gastric tube, and mice in LPS + OA‐rich EN groups received OA‐rich EN (OA dose: 0.25, 0.5 and 1.0 mL/kg/day, respectively) via the gastric tube. Following a 7‐day nutritional intervention period, mice in the Control group were intraperitoneally (i.p.) injected with saline, while mice in the other groups received an i.p. injection of LPS (5 mg/kg) to establish a sepsis model (Figure 1A).

Next, to upregulate GPR81 expression, CHBA was used as a specific GPR81 agonist. Mice were divided into four groups (*n* = 8 per group): Control, LPS, LPS + OA‐rich EN, and LPS + OA‐rich EN + CHBA. Mice in the Control and LPS groups were treated identically to those in the above experiment. Mice in the LPS + OA‐rich EN and LPS + OA‐rich EN + CHBA groups received OA‐rich EN (0.5 mL/kg/day) via the gastric tube for 7 days prior to LPS administration, and mice in the LPS + OA‐rich EN + CHBA group were additionally administered CHBA (50 mg/kg/day) via oral gavage (Figure 4A) (Ohno et al. 2023).

Furthermore, AAV9‐CTRP6 and AAV9‐MKK6 were used to specifically enhance CTRP6 expression and p38 activity, respectively. Mice were divided into five groups (*n* = 8 per group): Control + Vector, LPS + Vector, LPS + OA‐rich EN + Vector, LPS + OA‐rich EN + AAV9‐CTRP6, and LPS + OA‐rich EN + AAV9‐MKK6. Two weeks prior to gastric tube placement, mice in the Control + Vector, LPS + Vector, and LPS + OA‐rich EN + Vector groups were injected with the empty vector via tail vein, while those in the LPS + OA‐rich EN + AAV9‐CTRP6 and LPS + OA‐rich EN + AAV9‐MKK6 groups were administered AAV9‐CTRP6 and AAV9‐MKK6 via tail vein injection, respectively. Subsequently, mice in the Control + Vector and LPS + Vector groups were treated identically to the Control and LPS groups in the preceding experiment. The remaining three groups received OA‐rich EN (0.5 mL/kg/day) via the gastric tube for 7 days prior to LPS administration (Figure 6A).

All mice were maintained at a constant total caloric intake of 100 kcal/kg/day throughout the entire experimental period. Following LPS or saline injection, all mice continued to receive the corresponding nutritional intervention for 3 days. At the end of the experiment, all mice were humanely euthanized, and inguinal WAT, extensor digitorum longus (EDL), and serum samples were rapidly harvested. EDL was selected to assess sepsis‐induced skeletal muscle atrophy. Since EDL is a fast‐twitch glycolytic muscle highly susceptible to inflammatory and catabolic stress, and our previous study has confirmed that it exhibits marked atrophy in the acute phase of sepsis, accompanied by significant changes in the expression of atrophy‐related markers (Zhang et al. 2024).

### 2.5 Energy Expenditure (EE) Measurement

On the third day following LPS or sterile normal saline injection, mice were individually housed in metabolic cages (Columbus Instruments, CLAMS, USA), and EE was continuously recorded for 24 h under a standard 12 h light/12 h dark cycle.

### 2.6 Grip Strength Measurement

Mice were allowed to grasp the metal bar of a digital force gauge (Chatillon, model DFX II, USA) with their forelimbs and then were pulled horizontally backward by the tail at a constant speed until voluntary grip release. Each mouse was tested five times, with a 2‐min rest interval between trials to prevent fatigue. The final grip strength value was calculated as the mean of the five valid measurements.

### 2.7 WAT and Serum Lactate Measurements

The levels of lactate in serum and WAT were measured using a lactate assay kit. Before the measurement, WAT tissue was homogenized with lactate assay buffer at a ratio of 1 mg/0.1 g, followed by centrifugation at 12,000 *g* for 5 min at 4°C, and the supernatant was collected. Serum samples were used directly for measurement.

### 2.8 Serum β‐Hydroxybutyrate (β‐OHB) Measurement

The level of serum β‐OHB, the major ketone body, was measured using a ketone meter (Abbott, FreeStyle Optium Neo, UK).

### 2.9 Hematoxylin–Eosin (H&E) Staining

Fixed WAT and EDL tissues were embedded in paraffin, sectioned at 5 μm, and stained with H&E. Sections were examined under a light microscope and representative images were captured. The diameter of each adipocyte was measured using ImageJ software.

### 2.10 Immunohistochemical Staining

Paraffin sections of WAT were deparaffinized, rehydrated, and subjected to antigen retrieval in citrate buffer for 15 min. Endogenous peroxidase activity was blocked with 3% hydrogen peroxide for 10 min. Sections were incubated with the primary antibody against UCP1 (1:400) at 4°C overnight. After being washed with PBS, sections were incubated with the secondary antibody at room temperature for 1 h. Representative images were captured under a light microscope.

### 2.11 Western Blot

Proteins were extracted from WAT and EDL tissues. After being separated by sodium dodecyl sulfate‐polyacrylamide gel electrophoresis, proteins were transferred to polyvinylidene fluoride membranes. Membranes were blocked with 5% nonfat milk, followed by incubation with primary antibodies at 4°C overnight. Primary antibodies included: GPR81 (1:1000), CTRP6 (1:1000), p‐p38 (1:1000), p38 (1:1000), UCP1 (1:1000), and β‐tubulin (1:5000). Membranes were washed with TBST and incubated with secondary antibodies for 1 h at room temperature. Target proteins were detected using an ECL chemiluminescent reagent. Western blot band intensities were quantified using ImageJ software. GPR81, CTRP6, UCP1, MURF‐1, and MAFBx were normalized to the corresponding β‐tubulin, and p‐p38 was normalized to total p38. All the protein expressions were normalized to those of the control group.

### 2.12 Fasting Blood Glucose, Fasting Serum Insulin, and Insulin Resistance Measurements

Fasting blood glucose was measured using a glucometer (Roche, ACCU‐CHEK Performa, Switzerland) and fasting serum insulin was measured using an ELISA kit. The degree of insulin resistance was assessed using the homeostatic model assessment of insulin resistance (HOMA‐IR) index, calculated according to the following formula:

$$\begin{array}{c} \text{HOMA}‐\mathrm{IR}=\\ \frac{\text{fasting blood glucose}\;\left(\text{mmol}/L\right)\times \text{fasting serum insulin}\;\left(\mathrm{mIU}/\mathrm{mL}\right)}{22.5} \end{array}$$

### 2.13 Statistical Analysis

Statistical analyses were performed using GraphPad Prism (Version 10.4.2, GraphPad Software, Boston, Massachusetts USA). All data were expressed as the mean ± standard error of the mean (SEM). Differences among multiple groups were analyzed by one‐way analysis of variance (ANOVA) followed by Tukey's post hoc test. A *p*‐value < 0.05 was considered statistically significant.

## 3 Results

### 3.1 OA‐Rich EN Enhanced Ketone Body Synthesis and Decreased Lactate Accumulation in Serum and WAT of Septic Mice

To clarify the regulatory effects of OA‐rich EN on ketone body and lactate metabolism in septic mice, we detected the levels of serum β‐OHB, serum lactate and WAT lactate (Figure 1). Compared with the Control group, LPS‐induced sepsis led to a significant decrease in serum β‐OHB levels, while lactate levels in serum and WAT were markedly increased, indicating impaired ketogenesis and excessive lactate accumulation in septic mice. Conventional EN slightly increased serum β‐OHB levels and decreased lactate accumulation in serum and WAT, with no statistically significant differences observed. In contrast, OA‐rich EN intervention at different concentrations (0.25, 0.5, 1.0 mL/kg/day) dose‐dependently increased serum β‐OHB levels and decreased lactate levels in serum and WAT. Notably, the 0.5 mL/kg/day concentration exerted a robust and statistically significant regulatory effect, which was thus selected as the optimal concentration for subsequent mechanistic experiments. These results suggested that OA‐rich EN could effectively promote hepatic ketone body synthesis and suppress systemic and adipose tissue lactate accumulation in septic mice.

### 3.2 OA‐Rich EN Inhibited the GPR81‐CTRP6‐p38 Pathway and Suppressed WAT Browning in Septic Mice

**FIGURE 2:**
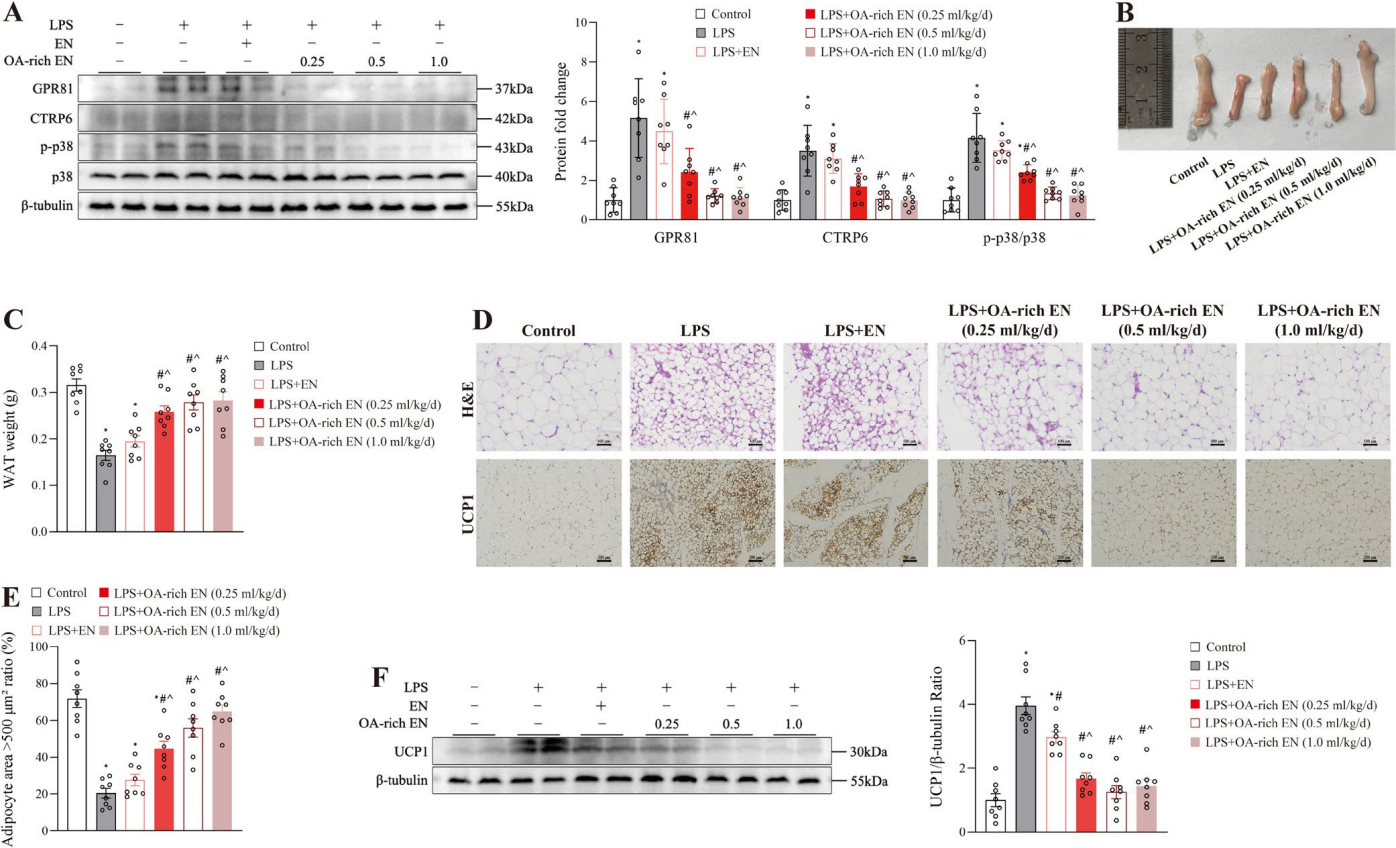
OA‐rich EN inhibited the GPR81‐CTRP6‐p38 pathway and suppressed WAT browning in septic mice. Western blot analysis of GPR81, CTRP6, p‐p38, and total p38 protein expression in WAT (A). Representative images of WAT (B). WAT weight (C). Representative H&E and immunohistochemical stained sections of WAT (D). Ratio of adipocytes with cross‐sectional area > 500 μm^2^ in WAT (E). Western blot analysis of UCP1 protein expression in WAT (F). *N* = 8 per group. Data are presented as the mean ± standard error of the mean. *p < 0.05 versus the Control group. #*p* < 0.05 versus the LPS group. ^*p* < 0.05 versus the LPS + EN group. CTRP6, C1q/tumor necrosis factor‐related protein 6; EN, enteral nutrition; GPR81, G‐protein‐coupled receptor 81; H&E, Hematoxylin–eosin; LPS, lipopolysaccharide; OA, octanoic acid; p‐p38, phosphorylated p38 mitogen‐activated protein kinase; UCP1, uncoupling protein 1; WAT, white adipose tissue.

We further investigated the effects of OA‐rich EN on the GPR81‐CTRP6‐p38 pathway and WAT browning in septic mice. Compared with the Control group, LPS significantly increased the protein expression of GPR81, CTRP6, and the phosphorylation level of p38 in WAT, indicating excessive activation of the GPR81‐CTRP6‐p38 pathway in septic mice. Conventional EN only slightly decreased the expression of the aforementioned proteins and p38 phosphorylation, with no statistical significance, whereas OA‐rich EN significantly inhibited LPS‐induced activation of the GPR81‐CTRP6‐p38 pathway (Figure 2A).

Morphological analysis and H&E staining of WAT revealed that LPS caused obvious WAT atrophy, reduced tissue weight and the formation of smaller multilocular adipocytes. Conventional EN exerted no significant protective effects against these LPS‐induced morphological abnormalities, while OA‐rich EN effectively restored WAT morphological structure and tissue weight, and increased the proportion of adipocytes with cross‐sectional area > 500 μm^2^ (Figure 2B–E). Similarly, LPS induced a significant increase in UCP1 expression in WAT. Conventional EN showed little effect on UCP1, while OA‐rich EN dose‐dependently decreased the overexpression of UCP1 (Figure 2D,F). These results suggested that OA‐rich EN could inhibit the GPR81‐CTRP6‐p38 pathway and suppress pathological WAT browning in septic mice.

### 3.3 OA‐Rich EN Alleviated Systemic Metabolic Disorders in Septic Mice

**FIGURE 3:**
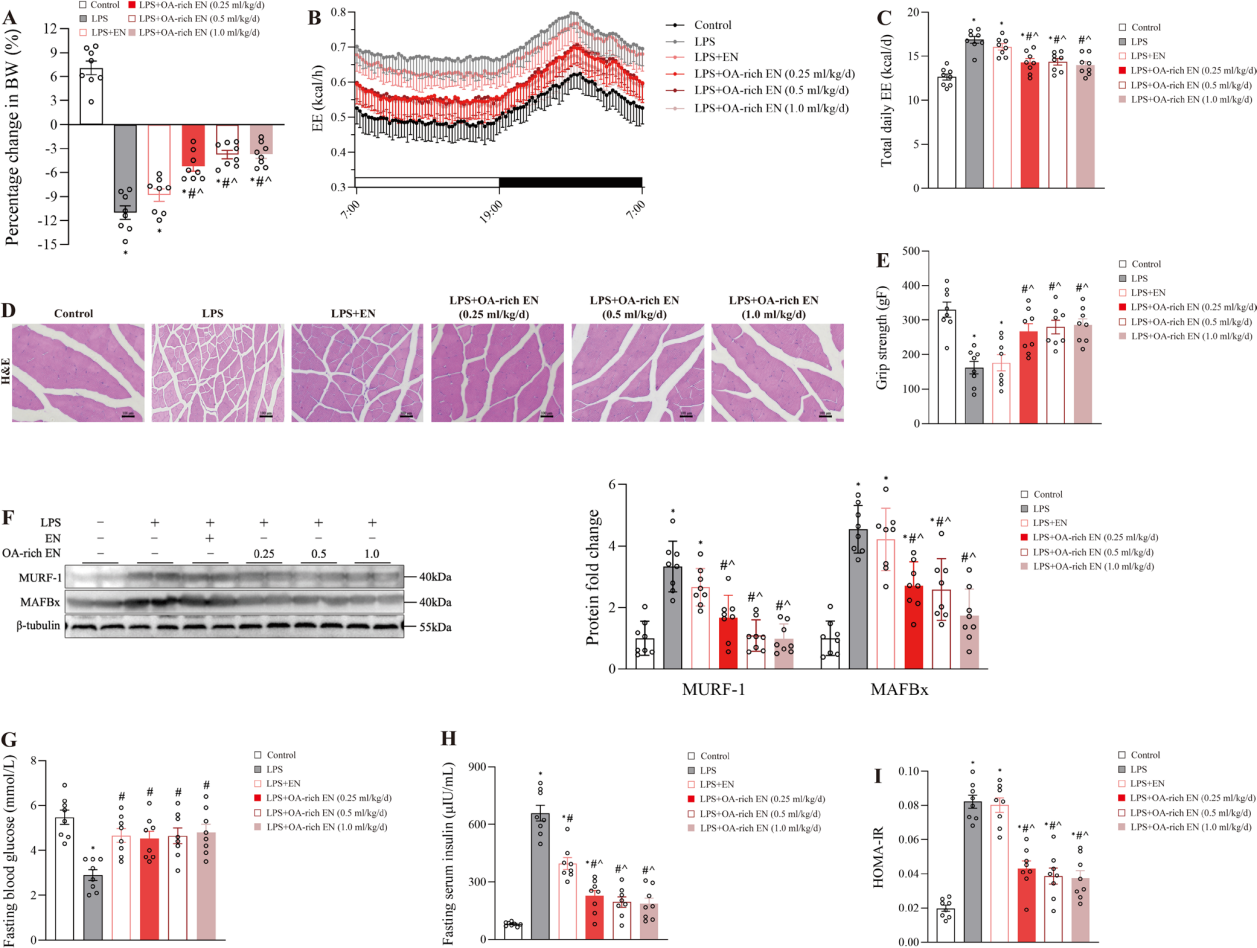
OA‐rich EN alleviated systemic metabolic disorders in septic mice. Percentage change in BW (A). Twenty‐four‐hour EE curve (B) and total daily EE (C) were measured using metabolic cages. Representative H&E stained sections of EDL (D). Grip strength (E) was measured using a digital force gauge. Western blot analysis of MURF‐1 and MAFBx in EDL (F). Fasting blood glucose (G) was measured using a glucometer. Fasting serum insulin (H) was measured by ELISA. The HOMA‐IR index (I). *N* = 8 per group. Data are presented as the mean ± standard error of the mean. *p < 0.05 versus the Control group. #*p* < 0.05 versus the LPS group. ^*p* < 0.05 versus the LPS + EN group. BW, body weight; EDL, extensor digitorum longus; EE, energy expenditure; EN, enteral nutrition; H&E, Hematoxylin–eosin; HOMA‐IR, homeostatic model assessment for insulin resistance; LPS, lipopolysaccharide; MAFBx, muscle atrophy F‐Box; MURF‐1, muscle ring‐finger‐1; OA, octanoic acid.

To evaluate the overall protective effects of OA‐rich EN on sepsis‐associated metabolic disorders, we assessed body weight change, total daily EE, skeletal muscle function, and insulin resistance in septic mice. Compared with the Control group, LPS led to a significant decrease in the body weight change and a significantly increased total daily EE, indicating the development of a severe negative energy balance in septic mice. Conventional EN only slightly alleviated the aforementioned abnormalities, while OA‐rich EN significantly reversed the body weight change and the excessive energy expenditure (Figure 3A–C).

Grip strength measurement showed that LPS caused a significant decline in the forelimb grip strength, and OA‐rich EN intervention significantly alleviated the skeletal muscle function in septic mice. H&E staining and detection of muscle atrophy markers in the EDL showed that LPS induced obvious EDL atrophy, while OA‐rich EN effectively reversed EDL morphological structure and inhibited the overexpression of muscle atrophy‐related markers (Figure 3D–F). With respect to insulin resistance, LPS induced a significant decrease in fasting blood glucose, accompanied by elevated fasting serum insulin levels and an increased HOMA‐IR index, indicating the development of insulin resistance in septic mice. Notably, conventional EN partially restored fasting blood glucose but failed to alleviate insulin resistance. In contrast, OA‐rich EN increased fasting blood glucose and decreased the HOMA‐IR index (Figure 3G–I). These results suggested that OA‐rich EN could effectively alleviate systemic metabolic disorders in septic mice, including negative energy balance, skeletal muscle dysfunction, and insulin resistance.

### 3.4 Activation of GPR81 Reversed the Protective Effects of OA‐Rich EN on WAT Browning and Metabolic Disorders in Septic Mice

**FIGURE 4:**
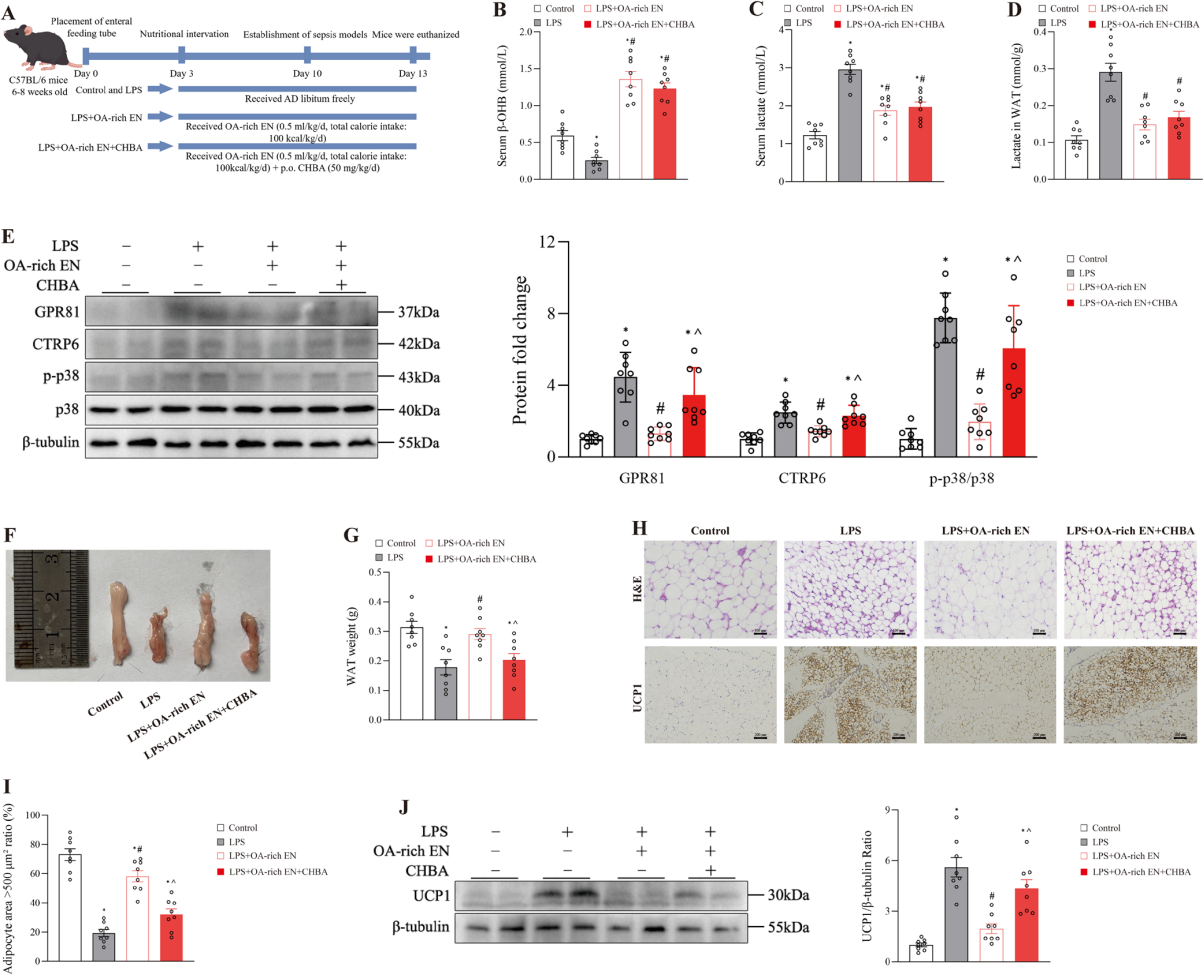
Activation of GPR81 reversed the protective effects of OA‐rich EN on the GPR81‐CTRP6‐p38 pathway and WAT browning without affecting ketogenesis and lactate metabolism in septic mice. Schematic illustration of the experimental design (A). Serum β‐OHB (B) was measured using a ketone meter. Lactate in serum (C) and WAT (D) were measured using a lactate assay kit. Western blot analysis of GPR81, CTRP6, p‐p38, and total p38 protein expression in WAT (E). Representative images of WAT (F). WAT weight (G). Representative H&E and immunohistochemical stained sections of WAT (H). Ratio of adipocytes with cross‐sectional area > 500 μm^2^ in WAT (I). Western blot analysis of UCP1 protein expression in WAT (J). *N* = 8 per group. Data are presented as the mean ± standard error of the mean. *p < 0.05 versus the Control group. #*p* < 0.05 versus the LPS group. ^*p* < 0.05 versus the LPS + OA‐rich EN group. CHBA, 3‐chloro‐5‐hydroxybenzoic acid; CTRP6, C1q/tumor necrosis factor‐related protein 6; EN, enteral nutrition; GPR81, G‐protein‐coupled receptor 81; H&E, Hematoxylin–eosin; LPS, lipopolysaccharide; OA, octanoic acid; p‐p38, phosphorylated p38 mitogen‐activated protein kinase; UCP1, uncoupling protein 1; WAT, white adipose tissue; β‐OHB, β‐hydroxybutyrate.

To verify whether GPR81 was the key target of OA‐rich EN in alleviating sepsis‐associated metabolic disorders, we upregulated GPR81 expression via CHBA (a specific GPR81 agonist) intervention in OA‐rich EN‐treated septic mice. Compared with the LPS + OA‐rich EN group, activation of GPR81 had no significant effect on serum β‐OHB levels or lactate levels in serum and WAT (Figure 4B–D), suggesting that OA‐rich EN regulates ketone body and lactate metabolism independently of GPR81.

Western blot analysis showed that activation of GPR81 significantly increased the protein expression of GPR81, CTRP6, and the phosphorylation of p38 in WAT, thereby reversing the inhibitory effect of OA‐rich EN on the GPR81‐CTRP6‐p38 pathway (Figure 4E). WAT morphological analysis, H&E staining, and UCP1 detection showed that activation of GPR81 reversed the protective effects of OA‐rich EN against WAT atrophy and UCP1 overexpression, thus reinducing pathological WAT browning in septic mice (Figure 4F–J).

**FIGURE 5:**
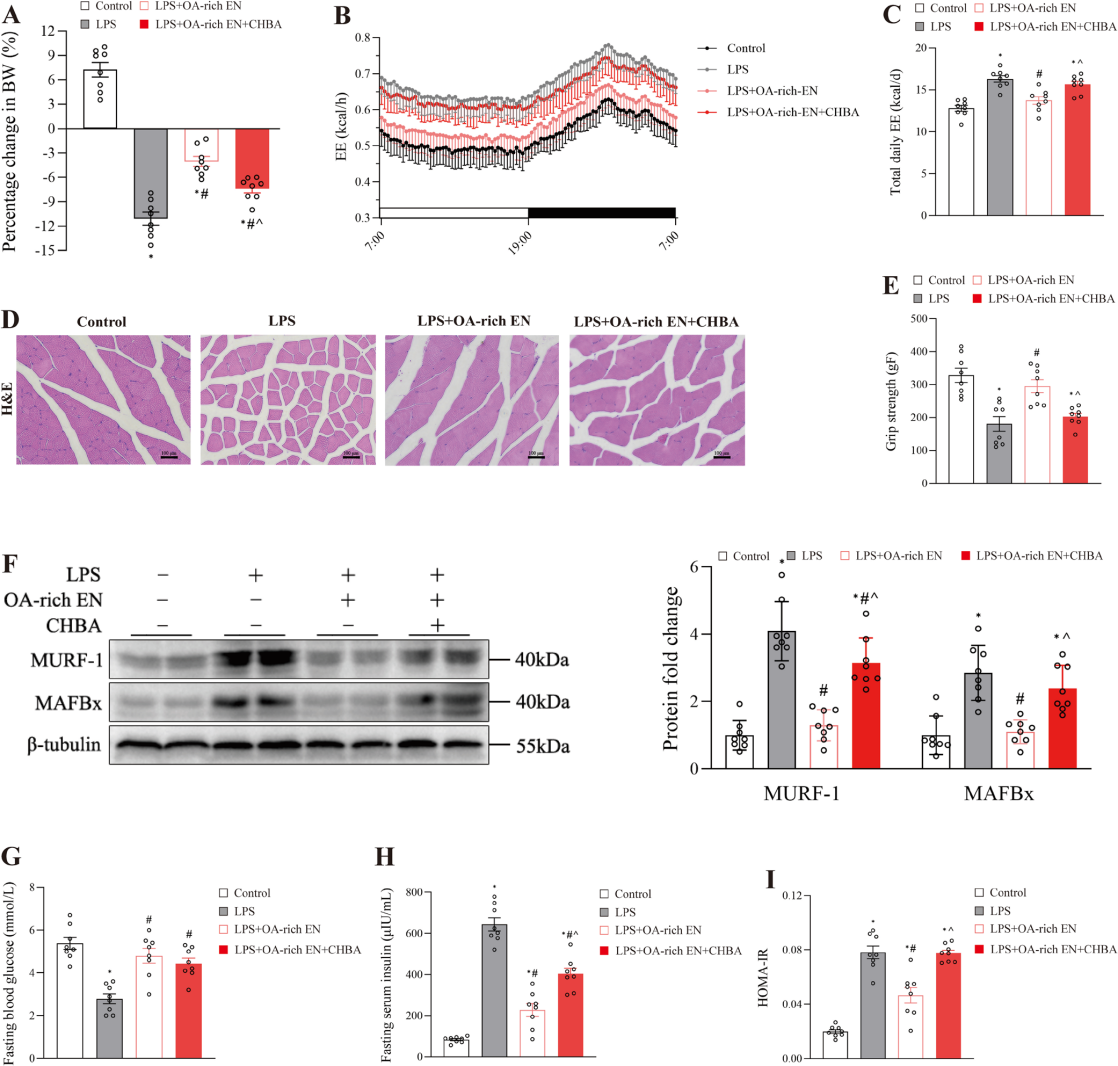
Activation of GPR81 reversed the protective effects of OA‐rich EN on systemic metabolic disorders in septic mice. Percentage change in BW (A). Twenty‐four‐hour EE curve (B) and total daily EE (C) were measured using metabolic cages. Representative H&E stained sections of EDL (D). Grip strength (E) was measured using a digital force gauge. Western blot analysis of MURF‐1 and MAFBx in EDL (F). Fasting blood glucose (G) was measured using a glucometer. Fasting serum insulin (H) was measured by ELISA. The HOMA‐IR index (I). *N* = 8 per group. Data are presented as the mean ± standard error of the mean. *p < 0.05 versus the Control group. #*p* < 0.05 versus the LPS group. ^*p* < 0.05 versus the LPS + OA‐rich EN group. BW, body weight; CHBA, 3‐chloro‐5‐hydroxybenzoic acid; EDL, extensor digitorum longus; EE, energy expenditure; EN, enteral nutrition; H&E, Hematoxylin–eosin; HOMA‐IR, homeostatic model assessment for insulin resistance; LPS, lipopolysaccharide; MAFBx, muscle atrophy F‐Box; MURF‐1, muscle ring‐finger‐1; OA, octanoic acid.

With respect to systemic metabolic disorders, activation of GPR81 significantly reversed the protective effects of OA‐rich EN on body weight change, excessive energy expenditure, grip strength decline, muscle atrophy, and insulin resistance (Figure 5). These results suggested that GPR81 was the core upstream target of OA‐rich EN in alleviating pathological WAT browning and sepsis‐associated metabolic disorders, and the protective effects of OA‐rich EN were dependent on the inhibition of GPR81 activity.

### 3.5 Activation of CTRP6 or p38 Reversed the Protective Effects of OA‐Rich EN on WAT Browning and Metabolic Disorders in Septic Mice

**FIGURE 6:**
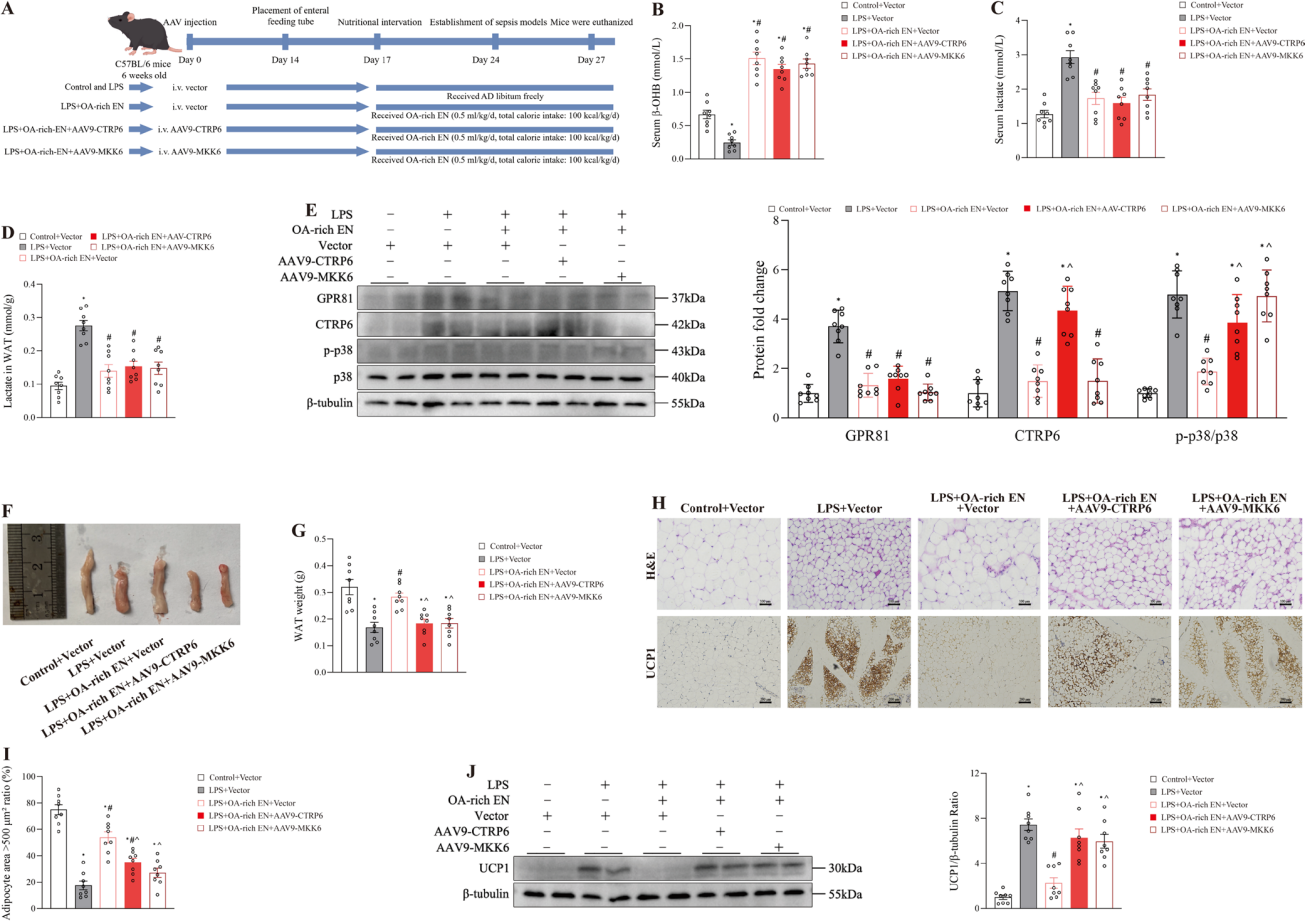
Activation of CTRP6 or p38 reversed the protective effects of OA‐rich EN on the GPR81‐CTRP6‐p38 pathway and WAT browning without affecting ketogenesis and lactate metabolism in septic mice. Schematic illustration of the experimental design (A). Serum β‐OHB (B) was measured using a ketone meter. Lactate in serum (C) and WAT (D) were measured using a lactate assay kit. Western blot analysis of GPR81, CTRP6, p‐p38, and total p38 protein expression in WAT (E). Representative images of WAT (F). WAT weight (G). Representative H&E and immunohistochemical stained sections of WAT (H). Ratio of adipocytes with cross‐sectional area > 500 μm^2^ in WAT (I). Western blot analysis of UCP1 protein expression in WAT (J). *N* = 8 per group. Data are presented as the mean ± standard error of the mean. *p < 0.05 versus the Control + Vector group. #*p* < 0.05 versus the LPS + Vector group. ^*p* < 0.05 versus the LPS + OA‐rich EN + Vector group. CTRP6, C1q/tumor necrosis factor‐related protein 6; EN, enteral nutrition; GPR81, G‐protein‐coupled receptor 81; H&E, Hematoxylin–eosin; LPS, lipopolysaccharide; OA, octanoic acid; p‐p38, phosphorylated p38 mitogen‐activated protein kinase; UCP1, uncoupling protein 1; WAT, white adipose tissue; β‐OHB, β‐hydroxybutyrate.

To further confirm that OA‐rich EN exerted its protective effects by inhibiting the entire lactate‐GPR81‐CTRP6‐p38 pathway, we specifically activated CTRP6 and p38 via tail vein injection of AAV9‐CTRP6 and AAV9‐MKK6, respectively, in OA‐rich EN‐treated septic mice. Compared with the LPS + OA‐rich EN + Vector group, specific activation of CTRP6 or p38 had no significant effect on serum β‐OHB levels or lactate levels in serum and WAT (Figure 6B–D), These results were consistent with those of the CHBA intervention, further confirming that OA‐rich EN regulated ketone body and lactate metabolism independently of the GPR81‐CTRP6‐p38 pathway.

Western blot analysis showed that activation of CTRP6 or p38 significantly increased the protein expression of CTRP6 and the phosphorylation of p38 in WAT, with no significant effect on GPR81 protein expression (Figure 6E). Morphological analysis, H&E staining, and UCP1 detection of WAT showed that activation of CTRP6 or p38 reversed the protective effects of OA‐rich EN against WAT atrophy and UCP1 overexpression, thus reinducing pathological WAT browning in septic mice (Figure 6F–J).

**FIGURE 7:**
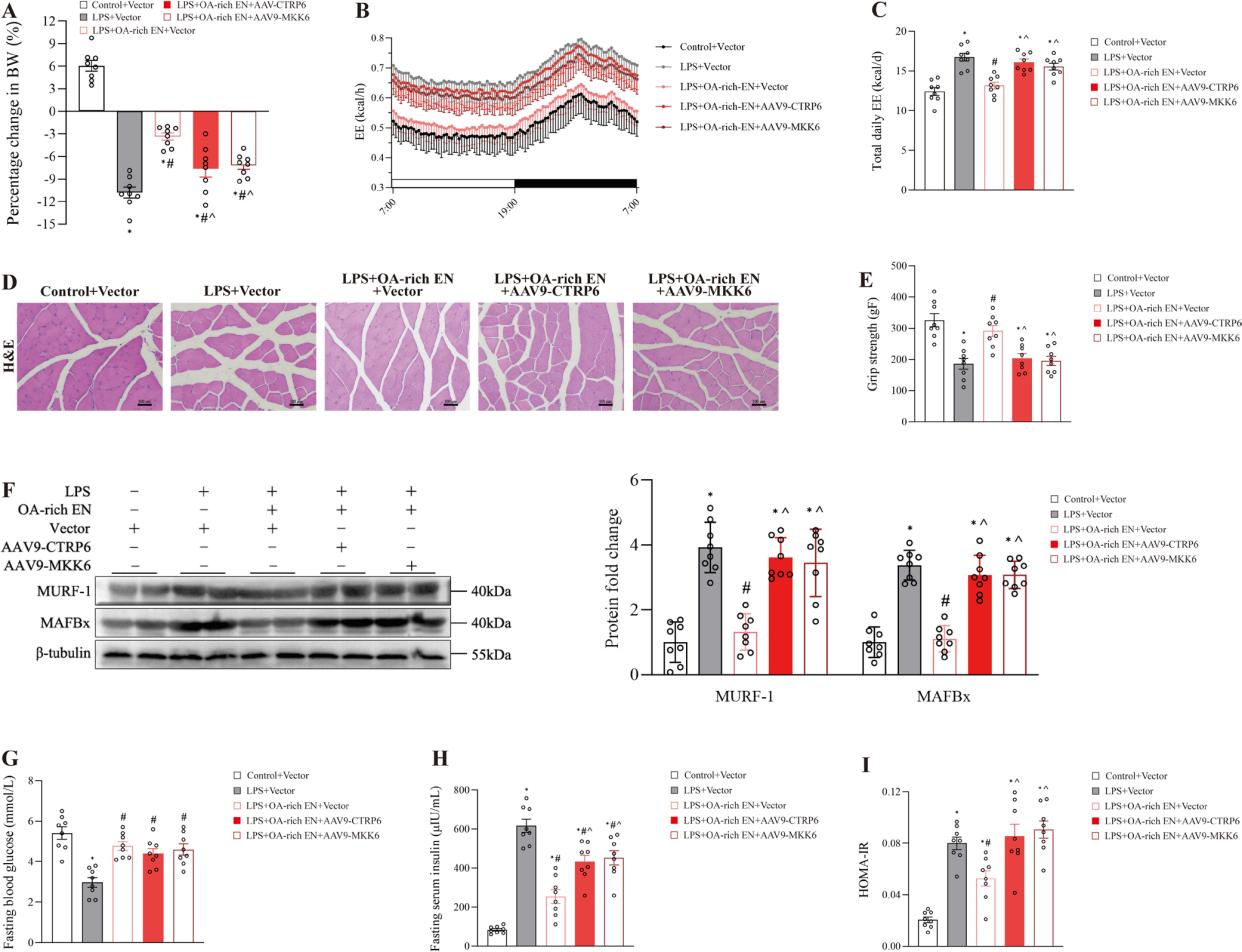
Activation of CTRP6 or p38 reversed the protective effects of OA‐rich EN on systemic metabolic disorders in septic mice. Percentage change in BW (A). Twenty‐four‐hour EE curve (B) and total daily EE (C) were measured using metabolic cages. Representative H&E stained sections of EDL (D). Grip strength (E) was measured using a digital force gauge. Western blot analysis of MURF‐1 and MAFBx in EDL (F). Fasting blood glucose (G) was measured using a glucometer. Fasting serum insulin (H) was measured by ELISA. The HOMA‐IR index (I). *N* = 8 per group. Data are presented as the mean ± standard error of the mean. *p < 0.05 versus the Control + Vector group. #*p* < 0.05 versus the LPS + Vector group. ^*p* < 0.05 versus the LPS + OA‐rich EN + Vector group. BW, body weight; EDL, extensor digitorum longus; EE, energy expenditure; EN, enteral nutrition; H&E, Hematoxylin–eosin; HOMA‐IR, homeostatic model assessment for insulin resistance; LPS, lipopolysaccharide; MAFBx, muscle atrophy F‐Box; MURF‐1, muscle ring‐finger‐1; OA, octanoic acid.

With respect to systemic metabolic disorders, activation of CTRP6 or p38 significantly reversed the protective effects of OA‐rich EN on body weight change, excessive energy expenditure, grip strength decline, muscle atrophy, and insulin resistance (Figure 7). These results further confirmed that OA‐rich EN exerted its protective effects by inhibiting the entire lactate‐GPR81‐CTRP6‐p38 pathway, and the activation of any key downstream molecule within this pathway could reverse the therapeutic effects of OA‐rich EN on pathological WAT browning and systemic metabolic disorders in septic mice.

## 4 Discussion

Sepsis is a life‐threatening clinical syndrome caused by a dysregulated host immune response to infection, with severe systemic metabolic disorders as a critical secondary complication that exacerbates disease progression and poor prognosis. And pathological WAT browning is a core event driving the development and progression of metabolic imbalance in septic hosts. The present study confirmed that OA‐rich EN exerts a prominent protective effect against metabolic disorders in LPS‐induced septic mice, and the core mechanism is that OA‐rich EN enhances hepatic ketogenesis to reduce lactate accumulation, thereby inhibiting the GPR81‐CTRP6‐p38 pathway and suppressing pathological WAT browning. Furthermore, specific activation of key molecules within this pathway can reverse the protective effects of OA‐rich EN, which clarifies the targeted nutritional regulatory role of OA‐rich EN in alleviating sepsis‐associated metabolic disorders and provides a novel theoretical basis for the optimization of clinical nutritional support strategies in septic patients.

Nutritional support is an indispensable cornerstone of sepsis therapy, and EN is the first‐line nutritional intervention for septic patients due to its advantages in preserving intestinal barrier integrity, regulating immune responses and reducing infectious complications (Wischmeyer et al. 2023). Conventional EN lacks targeted regulation of sepsis‐related pathological metabolism. It fails to correct severe disorders such as hypercatabolism and insulin resistance, limiting the efficacy of nutritional support (Niekerk et al. 2020). The key to addressing this clinical dilemma lies in developing optimized EN formulations based on the underlying mechanism of sepsis‐associated metabolic disorders and exploiting the specific nutritional regulatory properties of functional nutrients. OA, as a C8 MCFA with unique metabolic characteristics, has emerged as a promising functional nutrient for sepsis treatment (Tang et al. 2025). Unlike long‐chain fatty acids, OA is rapidly absorbed via the portal vein, directly undergoes hepatic β‐oxidation without the need for carnitine‐mediated transport, and exhibits the highest ketogenic efficiency among all MCFAs (Goossens et al. 2019; Vandenberghe et al. 2017). Ketone body metabolism is closely associated with lactate homeostasis in the septic state: serum ketone bodies levels are positively correlated with survival rates in septic patients, and exogenous ketone body supplementation can inhibit glycolysis and lactate production, thereby preventing sepsis‐induced muscle weakness (Acar et al. 2021; Goossens et al. 2019). As the MCFAs with the most potent ketogenic activity, OA is rapidly metabolized to ketone bodies in the liver following absorption, which could effectively inhibit the excessive lactate production and accumulation in septic hosts. The present study demonstrated that OA‐rich EN significantly promoted serum β‐OHB synthesis and effectively decreased lactate accumulation in serum and WAT in septic mice. This unique metabolic regulatory property of OA‐rich EN is of great clinical significance for septic patients with impaired glucose metabolism and insufficient energy supply, as ketone bodies can serve as an alternative energy supply for vital organs, thereby alleviating the energy deficit caused by sepsis (Soni et al. 2022).

Sepsis‐induced WAT browning is not an isolated pathological event, but is closely interconnected with the occurrence and development of multiple metabolic disorders: heat‐mediated energy dissipation caused by WAT browning exacerbates the hypermetabolic state and negative energy balance in septic hosts, which in turn promotes skeletal muscle proteolysis and ultimately leads to skeletal muscle dysfunction (Abdullahi and Jeschke 2017). The present study identified the lactate‐GPR81‐CTRP6‐p38 pathway as the key mediator of pathological WAT browning in sepsis and confirmed that this pathway is the core molecular target of the nutritional regulatory effects of OA‐rich EN. GPR81 is a lactate‐specific receptor predominantly expressed in adipose tissue, and excessive lactate accumulation in serum and WAT during sepsis is the initial trigger of pathological WAT browning: lactate binds to GPR81 to upregulate CTRP6 protein expression, which then activates p38 phosphorylation; phosphorylated p38 further promotes the nuclear translocation of ATF2, thereby upregulating UCP1 gene transcription and protein expression, and ultimately inducing the transformation of WAT into a brown‐like phenotype (Cao et al. 2004; Xu et al. 2024; Yao et al. 2020). OA‐rich EN exerts its nutritional regulatory effects by enhancing hepatic ketogenesis to decrease lactate accumulation, which directly inhibits the activation of GPR81 and the subsequent downstream signaling cascade, thus effectively alleviating systemic metabolic disorders induced by pathological WAT browning. Mechanistic verification in the present study further confirmed the specificity of this nutritional regulatory effect: GPR81 upregulation via CHBA (a specific GPR81 agonist) completely reversed the inhibitory effects of OA‐rich EN on the GPR81‐CTRP6‐p38 pathway and pathological WAT browning and simultaneously reversed the protective effects of OA‐rich EN on negative energy balance, skeletal muscle dysfunction and insulin resistance; specific activation of CTRP6 and p38 via AAV9‐CTRP6 and AAV9‐MKK6 also reversed the protective effects of OA‐rich EN on all metabolic disorder‐related indicators, with no significant effects on serum ketone bodies and lactate levels or GPR81 protein expression. These results fully confirmed that OA‐rich EN is not a simple energy‐supplementing nutritional formulation, but an optimized EN formulation with specific systemic nutritional regulatory effects via targeted inhibition of the GPR81‐CTRP6‐p38 pathway and that the regulation of ketone body and lactate metabolism by OA‐rich EN is independent of this pathway.

Notably, the present study further defines OA‐rich EN as a novel optimized EN formula with targeted systemic nutritional regulatory effects, which is fundamentally different from conventional EN that only provides basic energy and nutrient supplementation. In contrast, OA‐rich EN targets the lactate‐GPR81‐CTRP6‐p38 pathway to suppress pathological WAT browning. This strategy shifts sepsis nutrition from empirical supplementation toward mechanism‐guided targeted intervention. This characteristic endows OA‐rich EN with important clinical inspiration for the optimization of nutritional support strategies for septic patients: on the one hand, it provides a new idea for the development of functional EN formulations for critical illnesses, that is, the integration of functional nutrients with specific biological activities based on the core pathological links of sepsis to construct optimized EN with targeted regulatory effects; on the other hand, it advocates the concept of precision nutritional support for sepsis, and for septic patients with hyperlactatemia and obvious WAT browning, OA‐rich EN can be used as a targeted nutritional intervention to correct metabolic disorders at the molecular level while supplementing energy. In addition, the targeted regulatory effect of OA‐rich EN on the lactate‐GPR81‐CTRP6‐p38 pathway also indicates its promising translational potential beyond sepsis. It can be extended to other hypermetabolic critical conditions accompanied by pathological WAT browning, such as severe burns, cancer cachexia, and major trauma, which greatly expands the clinical translational value of this optimized EN formula.

It should be noted that the present study has certain limitations that warrant further investigation in subsequent studies. First, we used a 5 mg/kg LPS‐induced sepsis model rather than a cecal ligation and puncture (CLP) model. The LPS model was chosen because it induces a synchronized, reproducible systemic inflammatory and metabolic response, which is ideal for investigating early mechanisms of WAT browning and metabolic disorders. In contrast, the CLP model closely mimics clinical polymicrobial sepsis but exhibits high variability in infection severity, bacterial load, and mortality, which may confound the interpretation of metabolic outcomes. Additionally, LPS doses ranging from 1 to 10 mg/kg are commonly used in sepsis research (Marefati et al. 2023; Wang et al. 2024; Zhang et al. 2022), and 5 mg/kg is consistent with our previous work (Zhang et al. 2024), reliably inducing skeletal muscle atrophy and insulin resistance. Following gastric tube placement and surgical stress in all mice, our pilot data showed that LPS administration at 5 mg/kg resulted in nearly 10% 3‐day mortality, whereas 10 mg/kg LPS led to nearly 50% mortality. High mortality could mask nutritional intervention effects, so 5 mg/kg LPS was selected for a stable, low‐mortality model. However, the single‐hit LPS model has a simpler pathological process than clinical polymicrobial sepsis. Future studies using CLP or higher‐dose LPS models are needed to validate our findings in more clinically relevant settings. Second, the present study only investigated the effects of OA‐rich EN at three different concentrations on septic mice, and selected 0.5 mL/kg/day as the intervention concentration for subsequent mechanistic experiments based on its robust regulatory effects. The optimal clinical dosage, administration time, treatment duration, and delivery route of OA‐rich EN in clinical application remain to be elucidated through large‐scale preclinical animal experiments and well‐designed clinical trials.

## 5 Conclusion

In the present study, we demonstrated that OA‐rich EN exerted its protective effect against sepsis‐associated metabolic disorders by enhancing ketogenesis to suppress pathological WAT browning via targeting the lactate‐GPR81‐CTRP6‐p38 pathway. We further confirmed that OA‐rich EN was a novel optimized EN formulation with targeted systemic nutritional regulatory effects, which was significantly superior to conventional EN in alleviating sepsis‐associated metabolic disorders. This study enriched the understanding of the nutritional regulatory mechanism of OA in sepsis, identified the lactate‐GPR81‐CTRP6‐p38 pathway as a potential molecular target for the nutritional intervention against sepsis‐induced pathological WAT browning, and provided a promising targeted nutritional intervention strategy for the clinical nutritional management of septic patients with metabolic disorders. OA‐rich EN is expected to be translated into clinical practice as an optimized enteral nutrition formulation for sepsis treatment, which is significant for improving the efficacy of clinical nutritional support and the prognosis of septic patients with metabolic disorders.

## Author Contributions

**Chungen Xing:** conceptualization, funding acquisition, writing – original draft, writing – review and editing. **Chun Cao:** conceptualization, investigation, funding acquisition, writing – original draft, writing – review and editing, data curation. **Xiaohua Li:** methodology, investigation, writing – original draft, writing – review and editing, data curation. **Chunliang Zheng:** methodology, data curation, investigation, writing – original draft, writing – review and editing.

## Funding

This study was supported by the National Natural Science Foundation of China (82202369) and Jiangsu Key Laboratory for Organoid Creation and Precision Medicine (214267).

## Conflicts of Interest

The authors declare no conflicts of interest.

## Data Availability

The data that support the findings of this study are available on request from the corresponding author.
